# Assessing internal displacement patterns in Ukraine during the beginning of the Russian invasion in 2022

**DOI:** 10.1038/s41598-024-59814-w

**Published:** 2024-05-15

**Authors:** Yuya Shibuya, Nicholas Jones, Yoshihide Sekimoto

**Affiliations:** 1 The University of Tokyo, Tokyo, Japan; 2 World Bank, Washington DC, USA

**Keywords:** Human mobility, Ukraine, Internally displaced persons, Displacement, Turncated power law, Natural hazards, Civil engineering

## Abstract

Given the worldwide increase of forcibly displaced populations, particularly internally displaced persons (IDPs), it’s crucial to have an up-to-date and precise tracking framework for population movements. Here, we study how the spatial and temporal pattern of a large-scale internal population movement can be monitored using human mobility datasets by exploring the case of IDPs in Ukraine at the beginning of the Russian invasion of 2022. Specifically, this study examines the sizes and travel distances of internal displacements based on GPS human mobility data, using the combinations of mobility pattern estimation methods such as truncated power law fitting and visualizing the results for humanitarian operations. Our analysis reveals that, although the city of Kyiv started to lose its population around 5 weeks before the invasion, a significant drop happened in the second week of the invasion (4.3 times larger than the size of the population lost in 5 weeks before the invasion), and the population coming to the city increased again from the third week of the invasion, indicating that displaced people started to back to their homes. Meanwhile, adjacent southern areas of Kyiv and the areas close to the western borders experienced many migrants from the first week of the invasion and from the second to third weeks of the invasion, respectively. In addition, people from relatively higher-wealth areas tended to relocate their home locations far away from their original locations compared to those from other areas. For example, 19 % of people who originally lived in higher wealth areas in the North region, including the city of Kyiv, moved their home location more than 500 km, while only 9 % of those who originally lived in lower wealth areas in the North region moved their home location more than 500 km.

## Introduction

Forcibly displaced populations, such as refugees, asylum seekers, and internally displaced persons (IDPs), have drastically grown in the past decades due to war, poverty, and political and environmental factors, among others. According to the United Nations^1^, there were approximately 89 million of such population worldwide at the end of 2021, and IDPs were among the largest portion, 53.2 million people. As forcibly displaced populations face numerous challenges when seeking safety and security in new locations, the mobility patterns of IDPs have important implications for humanitarian response and policy makings. By understanding how IDPs move and where they go, aid organizations, local governments, and local communities can better distribute their resources and services. However, according to the United Nations^2^, many instances of internal displacement remain unrecorded. The absence or poor quality of data can leave hundreds of thousands of people without access to appropriate protection and assistance^2^. This led the United Nations to call for data and analysis to be conducted to recognize the realities of internal displacement and ensure durable solutions^2^.

In the past decade, the efforts to utilize human mobility GPS data for crises have been expanded. This includes measuring environmental exposure^3^, democratic participation (e.g., voter turnout^4^), disaster management (e.g., population displacement), and public health (e.g., spreads of diseases and non-pharmaceutical interventions for a pandemic^5–11^). Various studies have revealed that human movement is regular and predictable in normal times while some socio-economic and geographical variations exists^12–15^. On the other hand, despite massive efforts to leverage human mobility data collected from mobile phones for humanitarian aid, there has been less research on how human mobility data can aid IDPs.

**Figure 1:**
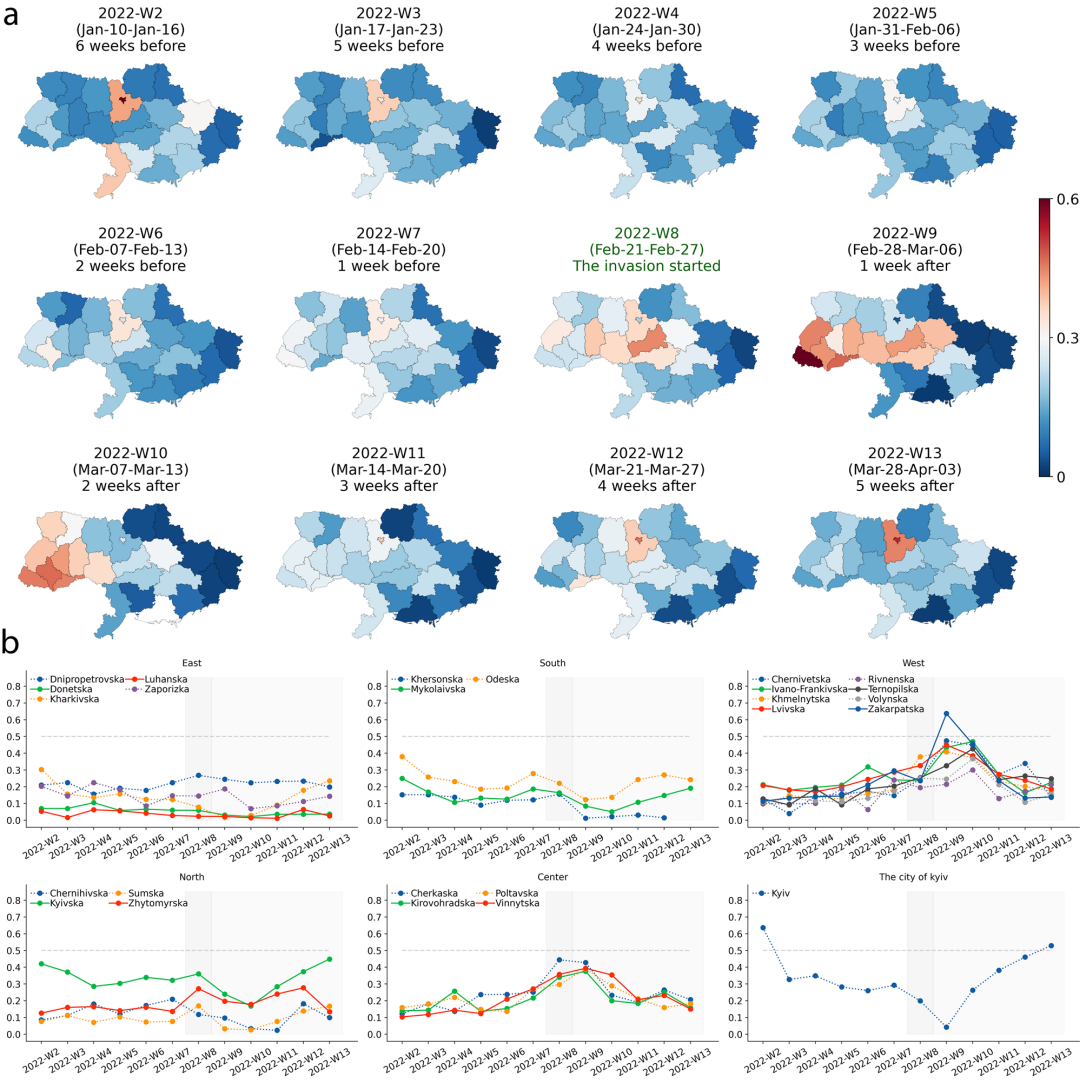
Move-out versus move-in population sizes of each oblast in each week (see section “Method”). In Panel (**a**), oblasts are colored redder(bluer) when a larger population moves into (out from) the oblasts relative to the moving-out (moving-in) population. 2022-Week-8 is when the invasion started. Panel (**b**) plots each week’s move-out versus move-in population for each oblast.

In the case of the IDPs during the Russian invasion of Ukraine, the International Organization for Migration (IOM) conducted telephone interviews in the early stages of the conflict^16^. In addition, scholars have leveraged social media data and satellite images to estimate displaced population movements^17–19^. Our analysis expands the previous efforts of IDP flow analysis by developing and showcasing a framework for tracking and visualizing up-to-date displaced population flows with fine-grained smartphone GPS data. Because one of the challenges of humanitarian aid with data is providing an up-to-date situation for the humanitarian stakeholders when they need the most^20,21^, our result visualizations can be used as basic information to detect areas with high humanitarian needs and assist the affected population. Furthermore, our analysis revealed correlations between socioeconomic levels and travel distances of IDPs. Because people’s decisions to relocate their settlements can depend on various socioeconomic factors, including access to transportation, finances, health, and family circumstances, among others, future research should keep investigate more on the drivers and barriers of displacements to support vulnerable populations when they need the help most.

**Figure 2:**
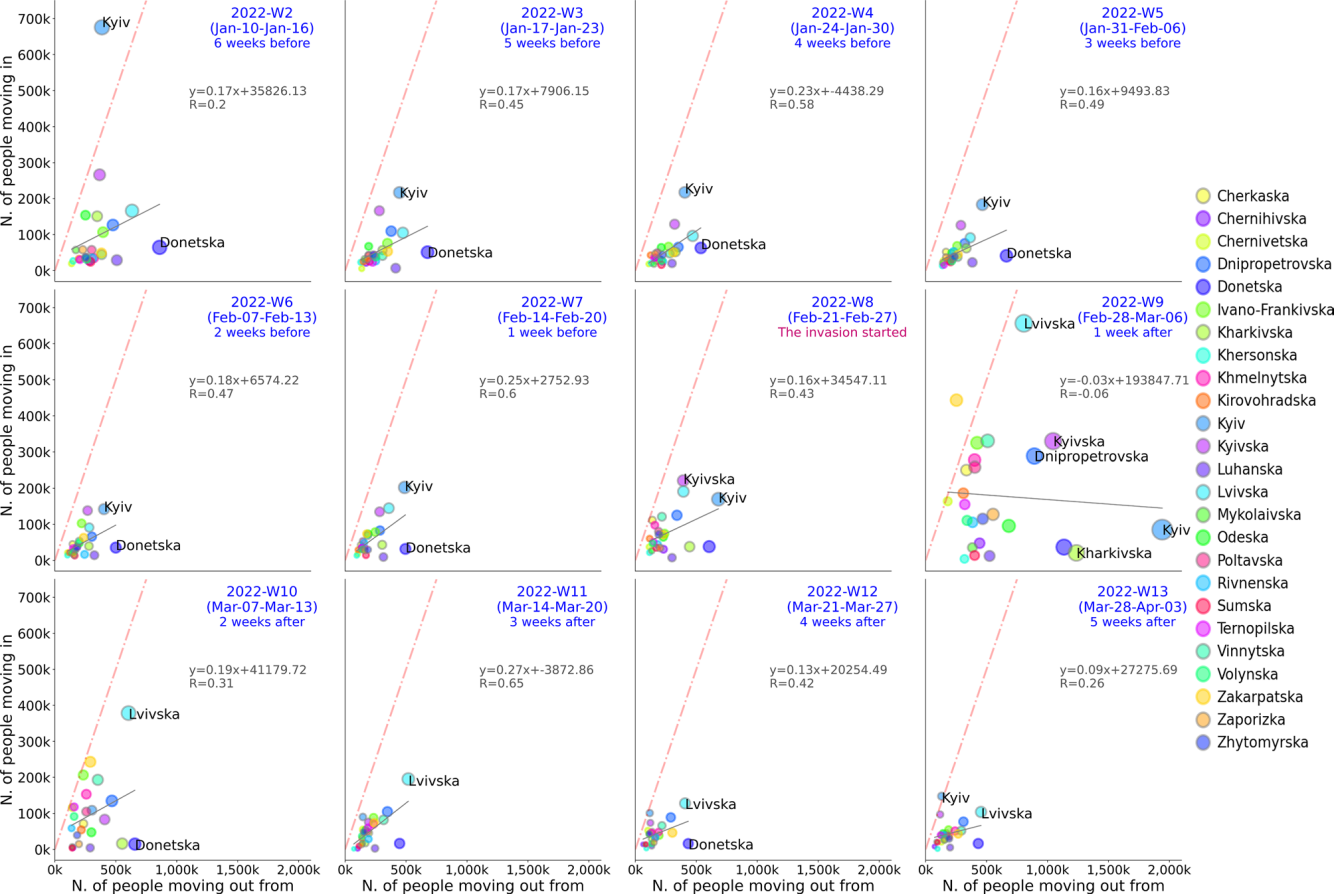
Estimated sizes of moving-out and moving-in populations of each oblast, where x-axis represents the number of people moving out from and y-axis represents the number of people moving in an area. The larger circles indicate larger population flows (sum of the both moving in and out). If a circle is on the dashed red line, which represents the linear line of $y=x$, it indicates that an oblast has an equal size of moving in and out populations. The grey solid line represents the regression line of the estimated moving out and moving in values. The estimated coefficients and correlation are shown in the upper right of each plot.

**Figure 3:**
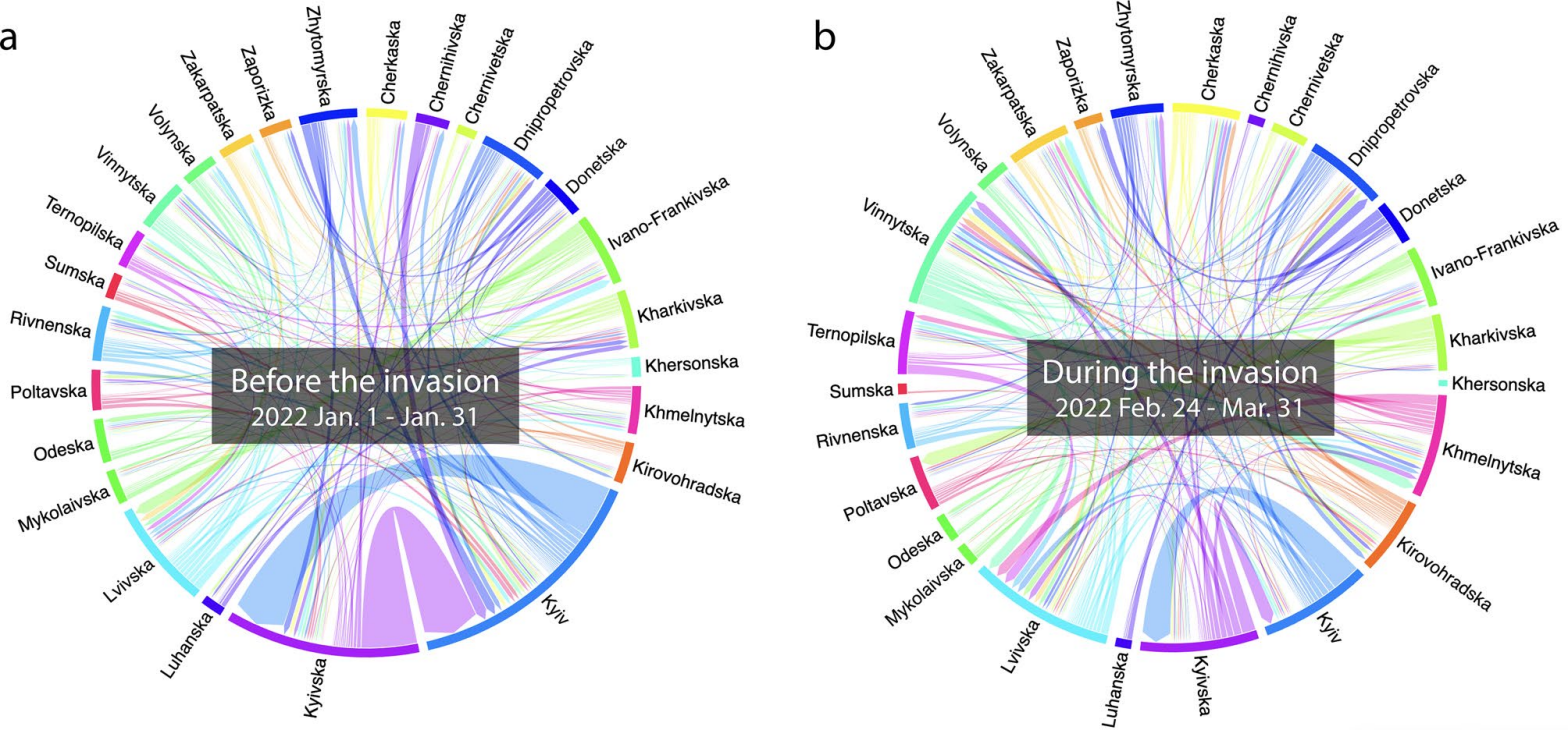
In-country human mobility connectivity among oblasts. Panel (**a**) shows the connectivity before the invasion (January of 2022), while Panel (**b** shows those during the invasion (from February 24 to March 31 in 2022). The arrows represent the human mobility directions. Note that the connectivity of oblasts with less than 10 times occurrences and the connectivity with outsider Ukraine nor unknown places were removed from these plots.

**Figure 4:**
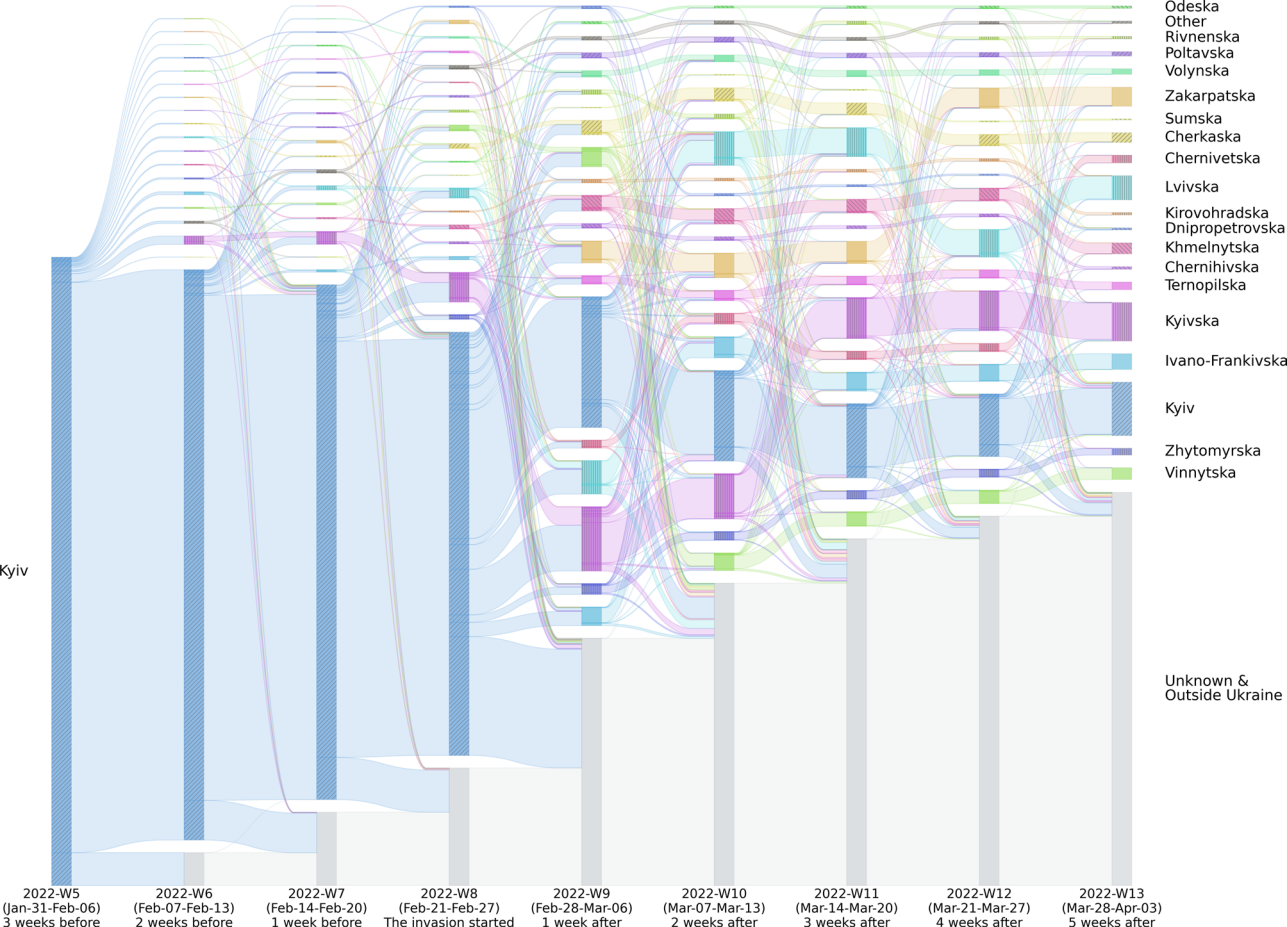
Estimated displaced population flow from the city of Kyiv. The bar heights represent the size of the population leaving oblasts and moving into oblasts. Note that the flows to “Unknown and Outside Ukraine” are, in particular, unclear because our datasets do not cover the flows outside Ukraine well.

## Results

Building upon the previous efforts to improve situational awareness with human mobility data, this study explores how IDPs’ initial occurrences can be captured with human mobility datasets by exploring the case of IDPs in Ukraine at the beginning of the Russian Invasion in 2022. To better understand human movements during the invasion, we use mobility containing periodic location updates containing timestamped geolocations (latitude, longitude, and time) provided by Outlogic (see section “Data description”). This dataset covers the period from January 1st to March 31st 2022 in Ukraine. We confine target populations to those who were in Ukraine in January 2022 and analyze their changes in every week’s home locations for a 3 month period that covers the beginning of the Russian invasion of Ukraine (February 24th, 2022). The data comprises over 9 million geographical records from over 139,761 unique anonymous users who opted in to share their location data anonymously (see section “Data description”).

In this paper, we focus on *sizes* and *distances* of internal displacements for the purpose of providing an initial assessment of IDP patterns. After checking our datasets’ data representativeness and overall quality from three perspectives (geolocation, time, and socioeconomic attributes) in Supplementary Material, we corrected our data with scaling factors based on the Ukraine population data of 2020 in Supplementary Material). We first estimate the sizes of the moving-in and the moving-out populations at the region level weekly by using a metric that identifies unusual patterns in the ratios of move-in and move-out population sizes to account for the lack of historical data in our datasets (see section “Method”). Secondly, we estimate each displaced person’s distance from the original home locations and check the variances of displacement distances with the truncated power law. Using the truncated power law model, we can capture the variance of human mobility patterns within and among regions, helping us understand geographical and socioeconomic differences in human mobility patterns (see section “Method”).

### Sizes of displaced population

To understand the overall flow sizes of the internally displaced persons, we estimate weekly home locations at the oblast level (see section “Method”), and then calculate each oblast’s relative move-in population by calculating the number of moving-in populations divided by the sum number of moving-out and moving-in populations per week (Fig. 1 and Supplementary Material). We consider the population who moved home location from an oblast to another oblast or unknown places including outside of the country as the move-out population while those who moved home location from another oblast to the oblast as the move-in population. In Fig. 1 Panel a, oblasts are colored redder when a larger population moves into the oblasts than moving-out population; oblasts are colored bluer when larger populations move out compared to moving-in populations. From the second week of 2022 (January 9th–15th, 5 weeks before the invasion started), the move-out populations were larger than the move-in populations in all oblast except the city of Kyiv. The east region’s oblasts had a larger move-out population even several weeks before the invasion started. When the invasion started (2022-Week-8), the divergence in the balance of oblasts’ move-in and move-out became larger. The city of Kyiv experienced a larger gap in move-out population relative to the move-in population, and Zakarpatska oblast experienced a larger move-in population compared to the move-out population (Fig. 1).

To further check the absolute sizes of the displaced populations of each oblast, Fig. 2 shows the scatter plot of the size of the population moving in (y-axis) and those moving out (x-axis). The population sizes of moving-in and moving-out started to become divergent around 2022-Week-8, while 2022-Week-9 (the following week of the invasion start) was the most divergent in the population flows. Some oblasts have lost much population (e.g., Kyiv, Kharikivska, Odeska, Donestska) while others have hosted more new populations, including Zakarpatska and Lvivska. The findings are consistent with previous research conducted on social media data^17,18^. They discovered that there were massive evacuations of major cities in the first few weeks of the conflict and that displaced persons moved from east to west during this time. In addition, to understand from and to where people moved in Ukraine, we created the displacement networks of oblasts by calculating the summed size of the displacement network *D* quantifies the number of trips from region *i* to *j*, recorded within a weekly time frame ($D_t^{ij}$). We created the aggregated sub-datasets consisting of the weekly home locations and plotted the displacement network flow of each sub-dataset (Fig. 3). To understand the case of population flows of Kyiv, Fig. 4 depicts the weekly displacement patterns among the persons whose estimated original home location was in Kyiv as of January 2022. This figure helps us grasp where the population moves weekly. According to Fig. 4, it appears that a certain portion of individuals frequently relocate their place of residence, while others remain in a single oblast. Note that the sizes of populations moving out outside Ukraine are possibly unreliable in our findings due to the lack of data coverage in the neighboring countries in our dataset.

### Travel distances of internally displaced populations

**Figure 5:**
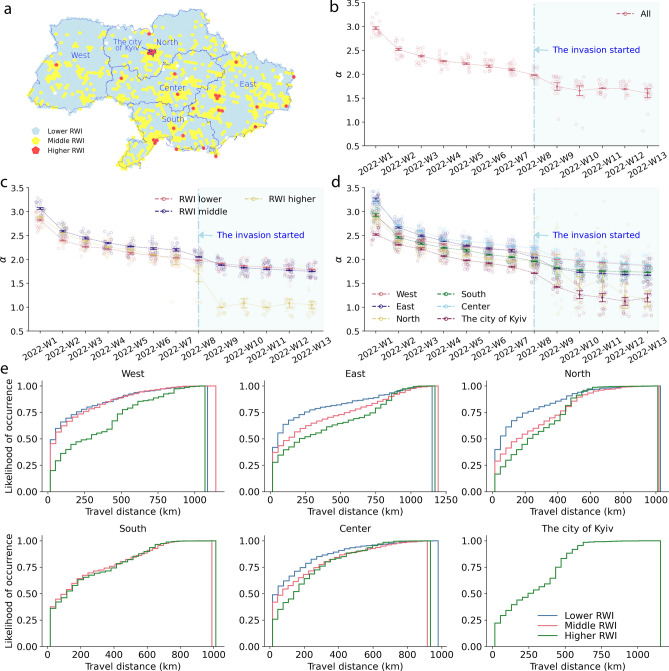
Travel distance trends. Panel (**a**) shows the geographical distributions of socioeconomic levels, Relative Wealth Index (RWI) (see section “Method”). ‘RWI Higher’: mean RWI $\ge 0.5$, ‘Middle RWI’: mean RWI $< 0.5$ and $> 0$, ‘Lower RWI’: mean RWI $\le$ 0). Panel (**b**–**d**) shows the estimated $\alpha$-values of truncated power law fittings ($p(x) \propto x^{-\alpha }e^{-\lambda x}$). 95 % percentile of mean values of estimated $\alpha$ with bootstrapping method are shown. A smaller $\alpha$ value signifies a more fat-tailed distribution, indicating larger differences in travel distances among populations. In other words, a smaller $\alpha$ indicates greater variation in travel distances. Panel (**e**) shows cumulative density distributions of travel distances of internal displacements for the whole dataset.

In addition to estimating the sizes of displaced populations shown above, we now shift our focus to examining the travel distances of internally displaced people. We assume that the travel distances of internally displaced persons may vary among individuals, depending on factors such as their original home locations, conditions in their surrounding areas and transportation accesses, and individual socioeconomic circumstances^20–24^. In this study, we compare travel distances among original home locations and regional socioeconomic levels by using the Relative Wealth Index (RWI) provided by Meta (see section “Method”). Typically, it is known that the frequency of human movement has been characterized as following a power law, wherein the probability of human movement decreases when the travel distance increases. These trends have been widely observed internationally, mainly during normal times^12,14,15,25^ and similar trends have been reported during emergencies, such as evacuation flows from disasters and during the pandemic^9,26^. Building upon previous research on human mobility in various countries, we employ a truncated power law with an exponential cutoff, $p(x) \propto x^{-\alpha }e^{-\lambda x}$). $\alpha$ is the scaling parameter and $\lambda$ is the parameter of the exponential distribution.

To verify the applicability of the truncated power law model for our dataset, we conducted the statistical test for checking the goodness of fit for the internal displacement flows in Ukraine (see section “Method”). The results indicated that the all week data at the country level follows the truncated power law distribution, and a majority of the macro-region level sub-dataset and RWI level sub-datasets also exhibit this pattern. Consequently, we decided to use the truncated power law for all datasets. To estimate the scaling parameter ($\alpha$), we employed a non-parametric bootstrapping method. A smaller $\alpha$ value signifies a more fat-tailed distribution, indicating larger differences in travel distances among populations. In other words, a smaller $\alpha$ indicates greater variation in travel distances. Panel b–d in Fig. 5 presents the estimated $\alpha$ values for the weekly travel distances from the original home location.

The results demonstrate that $\alpha$ values did not abruptly decline at the onset of the invasion. Instead, they gradually decreased from six weeks prior to the invasion. From 2022-Week-1 to 2022-Week-7 (before the beginning of the invasion), the estimated $\alpha$-values are between 2 and around 3 (Panel b in Fig. 5). These values are comparable to those reported in previous studies analyzing human mobility data^15^, where the means scaling parameters ($\alpha$) ranged from 1.80 in higher socio-economic areas to 2.54 in lower socio-economic areas. In the case of Ukraine, the estimated $\alpha$ value started to eclipse below 2 around the beginning of the invasion and experienced the sharpest decline in 2022-Week-9 (1 week after the beginning of the invasion). Subsequently, the $\alpha$ values remained low until the end of March 2022. Movement patterns were heterogeneous both under normal conditions, echoing results of previous human mobility studies during the crisis^26–28^. However, in the case of Ukraine, this heterogeneity became more pronounced following the onset of the invasion compared to normal times. When fitting the truncated power law to each RWI level group, we observed the largest drop in the estimated $\alpha$ values among the population originating from higher RWI areas (Panel c in Fig. 5). Conversely, the population from the middle and lower RWI areas experienced relatively moderate declines in the $\alpha$ values. This may be attributed to the higher perceived risks and the need to flee from the original locations among individuals in higher RWI areas, as well as their greater resources and capabilities to do so. Furthermore, we examined the distances to the western borders as a potential driver of people’s displacements by analyzing each macro-region displacement pattern and implementing the truncated power law fitting (Panel d–e in Fig. 5. The findings revealed that while displacement travel distance patterns vary between macro-regions, (Panel d in Fig. 5), they also differ within the same region depending on socioeconomic levels (Panel e in Fig. 5).

## Discussion

This study aims to improve the empirical understanding and immediate assessment of mobility patterns of internally displaced persons (IDPs) using human mobility data. With our proposed framework of analyzing and visualizing GPS human mobility data, we identified overall trends in internal displacement patterns while highlighting their heterogeneous characteristics. Our framework has the potential to be applicable in areas lacking historical human mobility data, facilitating immediate assessments even during a crisis. In addition, we observed that individuals from higher-wealth areas tended to relocate their locations far away from their original locations compared to those from other areas. Based on our results, we recommend further analysis of the heterogeneity of displacements as well as the drivers and obstacles behind displacement. In particular, given that not everyone can flee from crisis-affected places and those who are most vulnerable are usually poor^29^, investigating how different types of exposure to a crisis affect human behavior is crucial.

We believe that our methods can serve as an immediate situational check of IDPs. Nonetheless, for long-term aid for IDPs, more comprehensive and detailed analysis, as well as addressing potential data biases in human mobility data will be critical to fully utilize human mobility data benefits^13,30–35^.

## Method

### Data description

#### Human mobility data and home/stay points detection

To better understand human movements before and during the invasion, we use mobility containing periodic location updates containing timestamped geolocation (latitude, longitude, and time) provided by a provider of location data, Outlogic. We used the data collected in Ukraine from January 1st, 2022, to April 3rd, 2022, which covers the days from 6 weeks before the Russian invasion started (February 24th of 2022) (see Supplementary Material). We first apply a home detection method to location data points to infer weekly home locations per user. Specifically, we consider the most frequently occurring location (aggregated at a hexagonal grid, H3 indexing system resolution 5, https://h3geo.org/) per user per week from 9 pm to 5 am as the user’s home location at the oblast level. To ensure data quality and reduce privacy concerns, we only use aggregated levels of home location data in the analysis. The representativeness of the data has been tested and corrected with a scaling factor to reduce potential biases in our dataset (see Supplementary Material).

#### Geographic data

We use the oblast-level shape data provided by the Humanitarian Data Exchange (https://data.humdata.org/). The population of each oblast and H3 grid was calculated using the population distribution data sets from WorldPop (https://www.worldpop.org/).

### Descriptive characteristics of human mobility

The estimated weekly home locations are used to track how the population changed their home locations before and during the invasion. Considering the geographical representativeness of the data, the volume of people displaced from Kyiv to other areas has been plotted in Fig. 4, where the vertical length of bars represents the population volume. To understand the overall in-country displacement flows, we also use all oblast data to create the chord diagram in Fig. 2, where the weekly home locations are aggregated before and during the invasion time frame. In the chord diagram, we show the summed size of the displacement network *D* quantifies the number of trips from region *i* to *j*, recorded within a weekly time frame ($D_t^{ij}$).

### Displacement pattern detections

#### Relative frequency of moving-in to moving-out

The absolute size of the population moving into oblasts and the size of the population moving from oblasts are compared to understand the displacement trends (Fig. 1). We calculate the relative volume of the population moving in and out from oblasts, $RM_i^t$;

1 $$\begin{aligned} RM_i^t=I_i^t / \left( I_i^t+O_i^t \right) \end{aligned}$$

where *i* represent oblast *i*, and *t* represents week *t*, $I_i^t$ is the frequency of moving into the oblast *i* in a week *t*, and $O_i^t$ is the frequency of moving out from the oblast *i* in a week *t*. This simple metric can capture the relative frequency of population displacements at the oblast level. When $RM_i^t$ is close to 0.5, the frequency of occurrence of moving-in and moving-out is relatively similar. The frequency of occurrence of moving-in is higher when $RM_i^t$ is close to 1, while the frequency of occurrence of moving-out is higher when $RM_i^t$ is close to 0.

#### Travel distance analysis with the power-law model fitting

In addition to the size of the internally displaced population, we focus on the distance of internal displacement. Many human travel patterns have generally been known to follow the power law: in general, the probability of traveling to an area far away from one’s neighboring areas decreases with distance^36^. This trend is also observed in the human displacement patterns after a disruption in the areas^37^. By following previous human movement studies^12,15,38^, we use a simple model, the truncated power law, to fit the population’s home location change patterns.

With the maximum travel distances per person, we fit the following truncated power law with an exponential cutoff, where a probability density *p*(*x*):

2 $$\begin{aligned} p (x) \propto x^{-\alpha }e^{-\lambda x} \end{aligned}$$

where $\alpha$ is a constant scaling parameter and $\alpha >1$. $\lambda$ is the parameter of the exponential distribution. We use a maximum-likelihood fitting method with a goodness-of-fit test based on the Kolmogorov-Smirnov distance and likelihood ratio. The Kolmogorov-Smirnov distance is measured as the maximum distance between the empirical cumulative density function and the best-fit distribution’s cumulative density function. We also estimate the lower bound $x_{min}$ from which the data follow a power law, because, empirical data on human mobility, the data may only follow a power law from a lower bound $x_{min}$. We estimate the scaling parameter by numerically optimizing the log-likelihood.

3 $$\begin{aligned} \hat{\alpha } \approx 1 + n \left[ \sum _{i=1}^{n}ln \left( \frac{x_i}{x_{min}} \right) \right] ^{-1} \end{aligned}$$

where $x_i$, $i = 1 \, \ldots \, n$ are the observed values of x such that $x_{i} \ge x_{min}$. We apply Eq. (2) to the dataset of each week and estimate the weekly scaling parameter ($\alpha$, Fig. 5).

Even if our data are well fit by a power law, another distribution, such as an exponential or a log-normal, might still fit as well or better^39^. To ensure the validity of fitting the power law model, we compare the power law with alternative hypotheses via a likelihood ratio test, including exponential distribution. For each alternative, if the calculated likelihood ratio is significantly different from zero, then its sign indicates whether the alternative is favored over the power-law model or not.

To quantify the parameter uncertainty of the scaling parameter, we make use of the non-parametric bootstrap method. We drew points 999 times at random with replacement and estimated $x_{min}$ and $\alpha$ for each drawn dataset. By using the bootstrap method, the determination of displacement population sizes does not rely on any assumption of the statistical distribution. Another advantage of using the bootstrap method is the simplicity of implementation. This method enhances the applicability of population size estimations by relaxing the normality assumption.

#### Socioeconomic heterogeneity in population displacement

Whether a person can travel to secure places may depend on her geographical and socio-economic factors, including the accessibility to public transport, car owenership, finances and family situation. To capture such heterogeneity of travel patterns, we compare the distance from the displaced population’s original home location to the settled location with available geographical and socioeconomic attribute data (Fig. 5). Finally, we compare travel distance with the Relative Wealth Index. Because oblast is too broad to capture RWI’s geographical heterogeneousness, we use locations’ average RWI at the Hexigon area (H3, resolution 5).

### Supplementary Information

Supplementary Information.

## Data Availability

Human mobility data are only available for the current study. Geographic data (https://data.humdata.org/), population of each oblast (https://www.worldpop.org/) and Relative Wealth Index (RWI) provided by Meta (https://dataforgood.facebook.com/dfg/tools/relative-wealth-index) are publically accessible.

## Code availability

Python (version 3.8.11) was used to produce results for the analysis. Some custom code was developed for this article and will be available to the corresponding author by reasonable request.
