# Supplementary material for: Assessing internal displacement patterns in Ukraine during the beginning of the Russian invasion in 2022
Source: Sci Rep. 2024 May 15;14:11123. doi: 10.1038/s41598-024-59814-w (PMC11096167; doi:10.1038/s41598-024-59814-w)
Supplement: Supplementary file 1 — Supplementary Information. [file 41598_2024_59814_MOESM1_ESM.pdf]

# Supplementary Material for "Assessing Internal Displacement Patterns in Ukraine during the Beginning of the Russian Invasion in 2022"

**Yuya Shibuya et al.**[1,*]

[1]The University of Tokyo, Tokyo, Japan
[*]Corresponding author: yuya-shibuya@iii.u-tokyo.ac.jp

## S.1 Data quality check

### S.1.1 Data covarage

For the first step of our analysis, we check the representativeness of our datasets by focusing on (a) geographical boundaries, (b) time span, and (c) socioeconomic attributes (Fig. S.1). The underlying assumption for this step is that our data are missing some populations at random and not random. As human mobility data are passively collected by smartphones, they may have geographical and socio-economic characteristics compared to the actual population. This initial assessment helps us understand missing data mechanisms, possible relationships between measured variables, and the probability of missing data existing. In addition, this step enables us to ensure the analysis's validity and find a proper data aggregation level for further analysis. We found that more populated and socio-economically higher areas tend to have higher representativity in our dataset (Fig. S.1), echoing previously reported by related works [1]. To ensure our datasets are representative to the country's population, we corrected with a scaling parameter (see Section S.2). What follows here is the initial data quality check of our data before the data adjustment with a scaling parameter.

We first check the basic data statistics and data representativeness. To check whether samples hold reasonable spatial representation, we use the one-month data of January 2022 because more individuals must have displaced outside Ukraine after January. We also exclude users whose data were recorded only one day. We calculate the spatial representativeness by calculating the number of devices' home locations per population data sourced from WorldPop (Fig. S.1 Panel a) and compare the devices' home locations per population (Fig. S.1 Panel d) We also compare oblast-level population density and the samples (Fig. S.1).

### S.1.2 Compare the human mobility data with survey-based estimations

To further check if our data well represents the population movement in Ukraine, we compare the inner-nation displacement sizes with the survey-based IDP estimates published by the International Organization for Migration (IOM) (https://data.humdata.org/dataset/ukraine-idp-estimates). We used the estimation result of the Round 2 IDP Estimation. IOM data are only available at five macro-regions: West, East, North, Center, South, and the city of Kyiv. Thus, the comparison is conducted at the macro-region level (Fig. S.1 Panel b). In addition, we compare the population sizes moved from Ukraine between our data and the UN Refugee Agency (https://data.humdata.org/dataset/ukraine-refugee-situation). This analysis gave us substantial differences in the portion of human mobility from Ukraine to neighboring countries between our data and the data sourced from the UN Refugee Agency. Thus, we concluded that the human movement distilled by our data set is unsuitable for estimating refugees from Ukraine to neighboring countries.

### S.1.3 Temporal representativeness

We diagnose the temporal representativeness of our data by simply counting unique device numbers per day, how many days each device provides locational data, and how many pings they provide (recorded location data) are available for

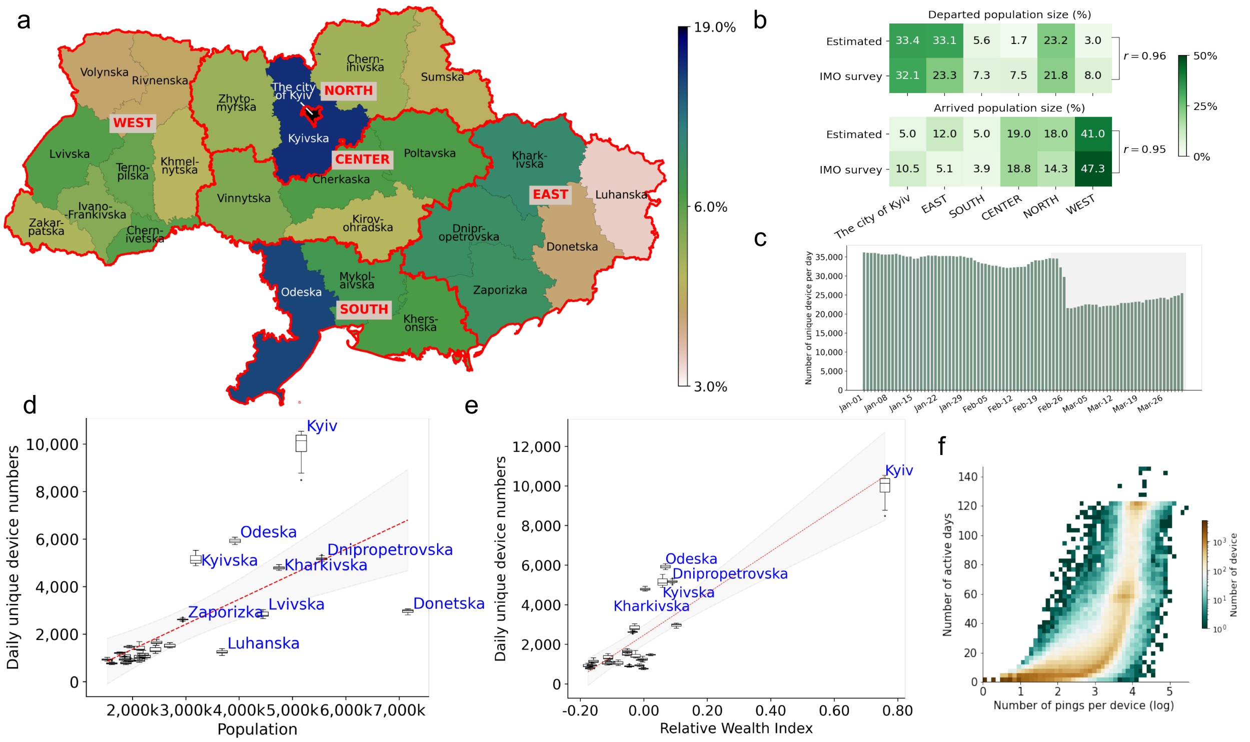


Figure S.1: The oblast-level geographical representativeness of the human mobility data (Panel a), the percentage comparison between IOM survey-based estimated IDPs and our human mobility data-based estimated IDPs (Panel b), the number of unique devices per day (Panel c), the correlation between the population density and average daily unique device number per area (Panel d), the socio-economic correlation with the human mobility data (Panel e), and 2D heat map of the number of pings and the number of active days per device (Panel f). The oblast-level colors describe the representativeness of our sample compared with the population. The map boundaries are drawn with the oblast-level shape files obtained from the Humanitarian Data Exchange (`https://data.humdata.org/`). The ratio of our sample per population density was calculated with the population data using 100 × 100 m population distribution data sets from WorldPop (`https://www.worldpop.org/`).

each device (Fig. S.1 Panel c and f) This assessment helps us to determine the appropriate date duration for the analysis (Fig. S.1 Panel c). In addition, to assess individuals' dropout rates during the time span, we check the number of active days per device, and the number of pings per user is examined (Fig. S.1 Panel f). We also check weekly percentage changes of population in Fig. S.2.

### S.1.4 Socioeconomic representativness

To consider the socioeconomic heterogeneity in the sample, we compare the device number per oblast and Relative Wealth Index (RWI) provided by Meta (`https://dataforgood.facebook.com/dfg/tools/relative-wealth-index`). We compare the daily unique device number at the oblast level and the RWI (Fig. S.1 Panel e) level. In doing so, we classify areas with higher, middle, and lower RWI and compare the IDP patterns among them.

## S.2 Population data adjustment with scaling factors

To ensure that the population in our GPS data is representative enough for the analysis, we calculated an adjusted population estimate using the scaling factor $\theta$.

$$\theta_i = \frac{N_i}{M_i} \tag{1}$$

$N_1$ represents the population of an oblast $i$ in 2020 while $M_i$ represents the population of January 2022 that is identified in our mobility GPS data. For all analysis, we calculated user $j$'s oblast-to-oblast movement with $\theta_i j$, that is, the user

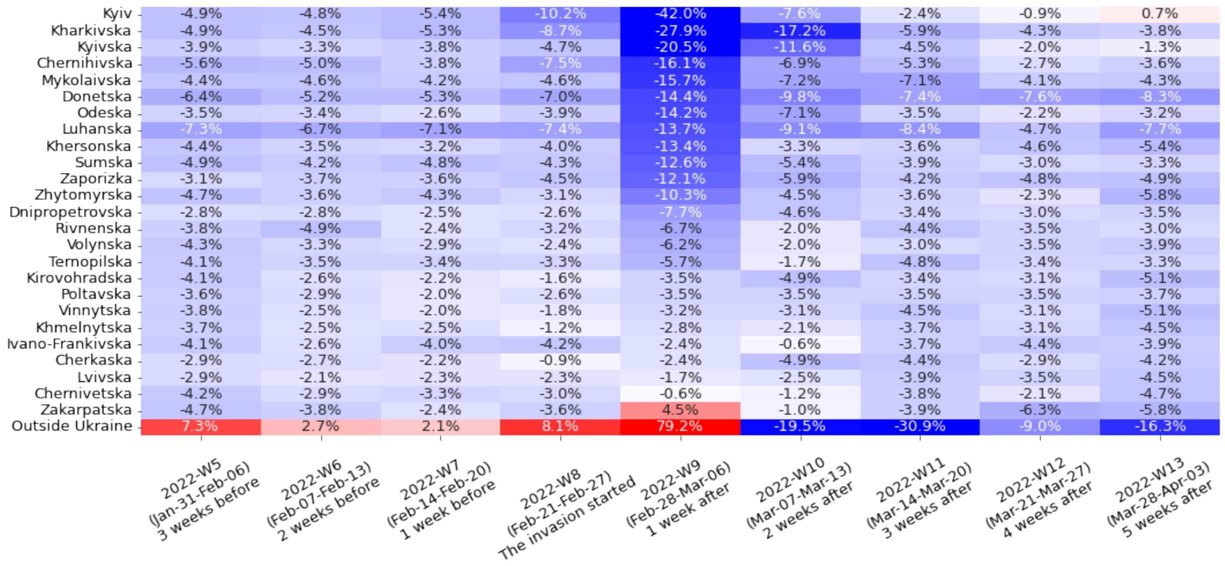


Figure S.2: Percent change of population from the previous week at each oblast level

$j$'s original home location $i$ in January 2020. This adjustment ensured that our adjusted estimates of current populations in each Oblast at the beginning of January 2022 summed to the total population of the open spatial demographic data. We have used WorldPop data of 2020 for the adjustment (`https://www.worldpop.org/`). All results in the main article are conducted with this adjusted population data.

## References

[1] Frank Schlosser, Vedran Sekara, Dirk Brockmann, and Manuel Garcia-Herranz. Biases in human mobility data impact epidemic modeling, December 2021. Number: arXiv:2112.12521 arXiv:2112.12521 [physics, q-bio].
